# Sleep initiation patterns and sleep quality among toddlers in the southeast of China: initial study results

**DOI:** 10.1186/s12887-024-04786-z

**Published:** 2024-05-03

**Authors:** Xiaoxia Lin, Xianrui Chen, Yanhui Chen, Ping Xu, Shan Chen

**Affiliations:** 1 Department of Pediatrics, Fujian Medical University Union Hospital, Fuzhou, 350001 China; 2 Department of Pediatric Rehabilitation, Xiamen Rehabilitation Hospital, No.468 Xianyue Road, Xiamen, 36100 China; 3 Fujian Family Planning Association, Fuzhou, 350001 China

**Keywords:** Sleep initiation pattern_1_, Sleep Quality_2_, Brief infant sleep questionnaire_3_, Toddler_4_

## Abstract

**Background:**

A large number of psychological consequences including sleep health emerged during the 2019 Coronavirus disease (COVID-19) pandemic. Sleep patterns in toddlers are vulnerable to negative environmental exposures, however, very few studies on this topic have been published so far.

**Objectives:**

In this paper, we aimed to investigate the sleep patterns and associated factors in toddlers from China confined at home in the context of COVID-19 pandemic.

**Methods:**

From April to November 2021, a convenience sample of 493 parents of young children aged (12–35 months) were surveyed from Fuzhou, Sanming, Quanzhou, Nanping, and Longyan cities in the Fujian Province, China. A cross-sectional survey was conducted via Electronic questionnaires to collect parent and child social-demographic characteristics. The Brief Infant Sleep Questionnaire (BISQ) was used to collect data on sleep practices, sleep duration and patterns, as well as the number of nocturnal awaking .

**Results:**

The mean age of toddlers was 2.11 years old, and 52.54% (259/493) were males. Among the 493 toddlers’ sleep patterns, 331(67.1%) initiated sleep accompanied by parents, 67(13.6%) slept independently, 59 (12.0%) were breast fed/bottle fed to initiate sleep, 27 (5.5%) were held and 9 (1.8%) rocked. The clear longitudinal association between the duration of night-time sleep, the frequency of nighttime awakenings, and various sleep patterns remains clear (*p* < 0.05). Multiple linear regression analysis indicated that sleep initiation with bottle-feeding/breast-feeding and rocked significantly increased the frequency of nighttime awakenings and reduced the duration of nighttime sleep (*p* < 0.05), as held was dramatically only for increasing the number of nighttime awakenings (*p* < 0.05). Multi-variate logistic regression analysis demonstrated that toddlers with severe sleep difficulties had a higher probability of being rocked to initiate sleep (*p* < 0.05). Conversely, young children with minor sleep problems were more apt to be in bed alone to initiate sleep (*p* < 0.05).

**Conclusions:**

During the COVID-19 pandemic, most infants and toddlers initiated sleep accompanied by parents and tend to have electronic media exposure before bedtime. Increased waking at night may be associated with sleep initiation with breast-feeding/bottle-feeding. Therefore, pediatric practitioners in primary community hospitals should pay attention to the education and promotion of sleep hygiene and parenting knowledge of young children to avoid the formation of poor sleep hygiene habits.

## Introduction

Infancy and early childhood play crucial roles in shaping sleep development and fostering the establishment of healthy sleep patterns and habits in children. A systematic review and meta-analysis showed that the prevalence of sleep problems for children in mainland China was 37.6% (95% CI:34.3–40.9%), with 38.9% in preschoolers and 33.3% in infants [1]. In 2008, Wang et al. [2] randomly selected 14,883 children aged 0–5 years from twelve cities in mainland China in a stratified manner. Of the objects,12.32% slept in a separate room by themselves, 25.30% slept in their own cribs put in parent’s room, and 62.48% took bed sharing with their parents. Among the three types of sleep arrangements, there were no significant difference in sleep duration at night. Nonetheless, when contrasted with the other patterns, children who co-sleep with their parents exhibited lower levels of autonomy, shorter durations of circadian rhythm sleep, reduced overall sleep time, and poorer sleep quality. Moreover, they displayed an increased likelihood of experiencing difficulties in falling asleep, frequent nighttime awakenings, and instances of bruxism. A stratified cluster random sampling method was used to sample 1,304 healthy full-term children at the age of 0–35 months from 8 provinces in China from 2012 to 2013, of whom 84.8% were bed-sharing, 13.2% were room-sharing, and 2.0% were the solitary sleep. A general linear regression analysis showed that bed-sharing was not statistically significant in predicting any sleep parameter(all *p* > 0.05) [3]. Bed-sharing is very common in Chinese infants aged 0–35 months, with a much higher prevalence than western counterparts. While bed-sharing demonstrates associations with the age of the infant and the family’s socioeconomic status, it is not hypothesized to have a direct effect on sleep outcomes [3]. In 2017, the “Guideline for Sleep Hygiene among Children Aged 0–5 Years” was published in mainland China [4], which has the following recommendations for family sleep parenting behaviors: Infants should sleep alone in their own cots, possibly in the same room as their parents, to develop their ability to fall asleep alone. Place them alone in a cot to sleep when they are drowsy but not asleep. It is not advisable to rock or snuggle the baby to sleep, and to separate feeding from sleeping.

Sleep patterns (the initiate sleep) has a certain impact on the sleep of infants and toddlers [5]. Liu et al. [6] performed a study in Southern China on 521 infants and toddlers aged 0–35 months, which revealed that 49.5% of the toddlers were breastfed/bottle fed to initiate sleep, 31.1% accompanied by their parents to initiated sleep, 11.9% were held/rocked to initiate sleep, and only 7.5% slept independently. During the COVID-19 pandemic, China implemented strict lockdown measures, including lockdowns of outbreak areas, restrictions on population movement, and traffic controls, effectively controlling the spread of the epidemic and safeguarding public safety and health. Liu et al. [7] conducted a study aiming to explore sleep patterns, sleep disturbances, and associated factors among Chinese preschoolers who were confined to their homes during the 2019 Coronavirus disease (COVID-19) outbreak. The researchers founded that compared to the 2018 sample, the preschoolers demonstrated changes in sleep patterns characterized by wake times and later bedtimes, shorter nap and longer nocturnal sleep duration, fewer sleep disturbances, and comparable 24-hr sleep duration reported by caregiver. A cross-sectional survey conducted in Spain during the COVID-19 pandemic (*n* = 254) showed that the proportion of parents who perceived their children to have problems sleeping was 39.4% and 44.1% (adjusted *p* = 0.363) before and under lockdown, respectively. The study indicates that home confinement generally has a negative impact on the infant’s and toddler’s sleep patterns [8]. Additionally, an online questionnaire-based study [9] showed that Japanese infants staying at home had significantly decreased percentage of outdoor play and total sleep time during the initial phases of the COVID-19 pandemic. Different forms of public health measures implemented by governments worldwide ranged from guidelines on physical distancing to mandates for individuals to stay at home. Although these measures are aimed at preventing the spread of Sars-CoV-2, they also have disrupted individuals’ everyday life tremendously and a negative impact on mental health [10]. Crying/sleeping problems were found in 13.5% of the infants, and noticeable behavior and emotional problems found in 8.5% of the toddlers. Overall, similar levels of mental health problems were observed in infants and toddler compared to pre-pandemic studies [7]. Nevertheless, there is currently limited evidence regarding the impact of toddlers’ sleeping patterns or sleep onset methods on their sleep quality in mainland China amidst the COVID-19 pandemic.

Early childhood health is a global public health issue worthy of attention. The long-term impact of the COVID-19 pandemic on early childhood development remains unclear. As China remains the largest developing country, it should actively explore avenues for knowledge and experience sharing with other countries (developing or developed), such as health development cooperation. In this paper, we investigated the sleep patterns and sleep hygiene status of toddlers from the southeast of mainland China confined at home during the period of COVID-19 pandemic, explored the factors influencing children’s sleep within this context, in order to provide a theoretical basis for promoting children’s health and informing intervention policies.

## Methods

### Sampling

From April to November 2021, the period of COVID-19 pandemic in the southeast of China, convenience sampling was used to select 544 Chinese origin families with a toddler age child from several community hospitals in Longyan, Nanping, Quanzhou, Sanming and Fuzhou cities in Fujian Province. During the data collection period, there were the confirmed cases of COVID-19 in the southeast of China. All parents of infants participating in the study granted informed consent. Ethical approval for our study was also obtained from the Scientific Research Ethics Committee of Fujian Medical University Union Hospital (2021KY131).

### Participants

(1) Inclusion: Healthy toddlers aged 12–35 months and their parents were included. (2) Exclusion: ① Toddlers with psychobehavioral developmental abnormalities, oncological diseases, rheumatological and immune diseases, or the use of medications known to cause sleepiness (e.g., antihistamines, anti-epileptics, and benzodiazepines) were excluded. ② Young children with developmental, mental, or physical disabilities, or receiving medications(e.g., antiepileptic drugs, antihistaminic drugs, glucocorticoids, and melatonin) were excluded, as well as these factors may affect sleep architecture including non-rapid eye movement (NREM) sleep and sleep cycles of rapid eye movement (REM).

### Measures

We interviewed participants face-to-face and collected all responses digitally. Caregivers completed the Chinese version of the Brief Infant Sleep Questionnaire (BISQ) [11, 12] including total sleep time (TST), number and timing of night wakings, sleep latency, sleep patterns, and any sleep problems over the past two weeks. The participants were also required to complete social-demographic characteristics (gender, age, residence, annual household income, room-sharing, caregiver education, parenting style, feeding method, etc.). The reliability and validity of the BISQ consists of 11 general questions have been previously verified, which is well for screening sleep problems for 0- to 3-years-old children. Total 24 h sleep duration, nighttime sleep, daytime sleep, and sleep initiation patterns were chose for sleep variables to assess sleep quality and quantity in toddlers [13].

### Statistical analysis

Data were tested by EpiData dual entry parallel consistency test. SPSS 22.0 software (Statistical Package for Social Sciences, Inc., Chicago, IL) was performed for statistical analysis. Excel 2003 was also used for data sorting and statistical analysis. Measurement data were expressed as mean ± standard deviation (SD), and qualitative data of categorical variables were presented as percentage and frequencies (%). The Student’s t-test of two independent samples for the comparison of continuous variables with normal distribution was used. Chi-square test was used for classification data. One-way analysis of variance (ANOVA) analyses was used for in-group evaluation and comparisons between the different groups. A multiple linear regression analysis was performed to evaluate the influence of sleep quality. The influencing factors of sleep initiation patterns were evaluated by a multivariate logistic regression analysis. All described results were based on an alpha level of 5%, which was considered statistically significant.

## Results

**Fig. 1:**
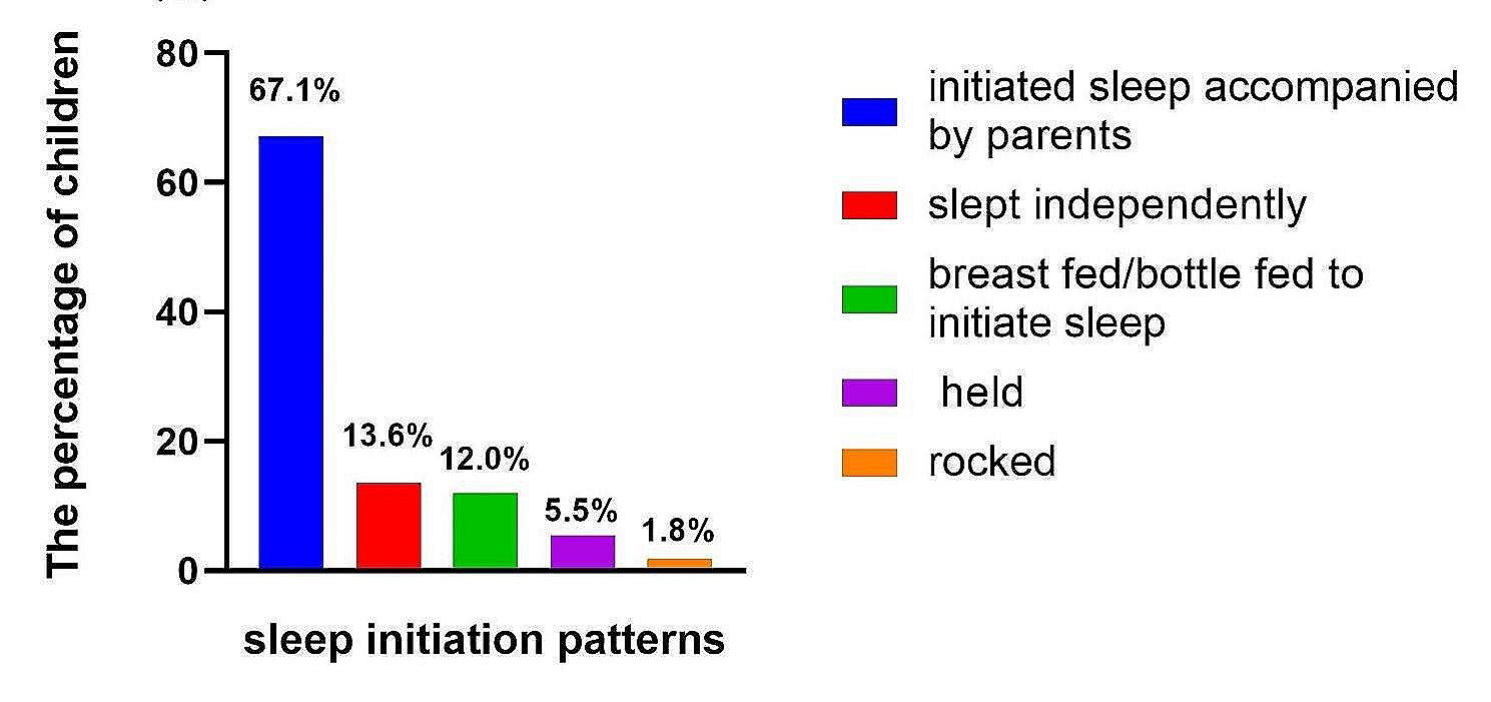
The frequency of toddlers’ sleep initiation patterns during COVID-19 pandemic reported by parents

Among the 544 surveyed families, there were 493 toddlers’ mothers approached and completed questionnaires, with a response rate of 90.66%. And 25 participants reported toddlers’ total sleep time was less than 4 h, and 16 participants reported toddlers’ total sleep time was more than 22 h. Based on previous literature, total sleep time more than 22 h or less than 4 h were considered extreme or inappropriate data which was considered that the mothers did not do the questionnaires carefully or do wrongly, and these questionnaires were invalid. Therefore these were excluded [11]. These excluded data didn’t affect the results. The mean age of toddlers was 2.11year-old. 52.54% (259/493) were males. An annual family income of toddlers less than 10,000 RMB was reported 9.7%, 10,000–50,000 RMB (25.4%), 50,000- 100,000 RMB (33.7%) and more than 100,000 RMB 31.2%. Among the 493 infants and toddlers, 331(67.1%) initiated sleep accompanied by parents, 67(13.6%) slept independently, 59 (12.0%) were breast fed/bottle fed to initiate sleep, 27 (5.5%) were held and 9 (1.8%) rocked(Fig. 1). Total 24 h sleep duration for all toddlers was 12.56(SD = 2.78) hr. Mean nighttime and daytime sleep duration of toddlers was 9.59 (SD = 1.65) hr and 2.96 (SD = 2.52) hr.

The results identified 5 types of Chinese toddlers’ sleep initiation patterns, as well as the significant differences in different annual household income (χ^2^ = 33.923, *p* = 0.001) and housing per capita area (χ^2^ = 20.265, *p* = 0.006). Screen time on different devices (such as TV, IPAD, mobile phone) (min), sleeping arrangement and sleep difficulties of toddlers were associated with sleep patterns (*p* < 0.05). There was no statistically significant correlation between toddlers’ sleep patterns and the mother’s age, maternal education and occupation, residence, watching TV in the bedroom, maternal age (at the time of the child’s birth), and parenting experience (first-time caregiver) and children’s gender, age, children’s daily outdoor activity time (min), children’s sleep position, feeding patterns, and bedtime routine(*p* > 0.05) (Table 1).

**Table 1:** Summary of outcomes by toddlers’ sleep patterns during the period of COVID-19 Pandemic

Item	Sample, n	While feeding	Being rocked	Being held	In bed alone	In bed near parents	F/χ^2^	*p*
Age of mother								
< 25 years old	10	0	0	0	5	5	9.039	0.102
25–35 years old	468	58	9	26	61	314		
> 35 years old	15	1	0	1	1	12		
Maternal education								
Primary school and below	14	1	1	2	4	6	23.415	0.103
Union high school	157	21	3	9	30	94		
Senior high school	109	12	2	2	14	79		
Bachelor’s degree	204	25	3	13	19	144		
Postgraduate or above	9	0	0	1	0	8		
Maternal occupation								
Stay-at-home Mom	171	29	5	8	24	105	9.003	0.307
Working	279	26	4	16	36	197		
Part-time	43	4	0	3	7	29		
Annual family income								
< 10,000 RMB	48	5	2	5	14	22	33.923	0.001
10,000 ~ 50,000 RMB	125	21	6	7	13	78		
50,000 ~ 100,000 RMB	166	19	0	8	24	115		
> 100,000 RMB	154	14	1	7	16	116		
Residence								
Rural	173	45	7	18	48	202	7.37	0.114
Urban	320	14	2	9	19	129		
Housing per capita area(m^2^)								
< 10	23	5	3	2	2	11	20.265	0.006
10 ~ 30	255	37	2	15	31	170		
> 30	215	17	4	10	34	150		
Parenting experience (first-time caregiver)								
Stern	34	4	0	2	8	20	10.905	0.175
Natural	178	30	4	11	23	110		
Multiple approaches	281	25	5	14	36	201		
Watching TV in the bedroom								
No	450	55	7	25	58	305	4.725	0.273
Yes	43	4	2	2	9	26		

One-way ANOVA analyses showed significant differences in parent-reported mean Daytime sleep duration (*p* = 0.001) and parent-reported mean number of nocturnal awakenings (*p* < 0.001) across all sleep patterns, although there was a dramatically increase in the mean Number of nocturnal awakenings from While feeding (2.39 ± 2.79; *p* < 0.001) to Being held (1.56 ± 0.97; *p* = 0.001). Compared to toddlers in bed alone, toddlers with while feeding had more daytime sleep (*p* = 0.001). No significant difference was detected in Nocturnal sleep duration, Nocturnal waking time and Night sleep latency (All *p* > 0.05) (Table 2).

**Table 2:** Comparison of sleep quality across all sleep patterns

	Cases (*n*)	Nocturnal sleep duration	Daytime sleep duration	Number of nocturnal awakenings	Nocturnal waking time	Night sleep latency
While feeding	59	9.38 ± 1.51	4.09 ± 2.99*	2.39 ± 2.79*	0.57 ± 0.94	0.77 ± 0.68
Being rocked	9	9.52 ± 3.05	3.90 ± 3.23	2.00 ± 0.87*	0.32 ± 0.22	1.04 ± 1.58
Being held	27	9.56 ± 1.81	3.51 ± 1.91	1.56 ± 0.97*	0.84 ± 1.32	0.62 ± 0.42
In bed near parent	331	9.56 ± 1.51	2.74 ± 2.35	0.82 ± 0.84	0.65 ± 1.44	0.78 ± 0.91
In bed alone	67	9.59 ± 1.65	2.76 ± 2.71	0.64 ± 0.60	0.64 ± 1.43	0.63 ± 0.74
F		1.157	4.466	24.469	0.303	0.832
*p*		0.329	0.001	< 0.001	0.876	0.505

*Compared with in bed alone, *p* < 0.05

Multivariable linear regression showed a difference in number of nocturnal awakenings between sleep initiation with bottle feeding/breast feeding and the other sleep patterns when adjusted for sleep patterns, sleep problem, residence, housing per capita area(m^2^), annual family income, screen time on different devices (min). Toddlers initiated sleep accompanied by parents and slept independently had a 1.5-time less than toddlers breast/bottle fed to initiate sleep (*p* < 0.001). Toddlers held to initiate sleep had a 0.5-time less than toddlers breast/bottle fed to initiate sleep (*p* < 0.001). Toddlers initiated sleep accompanied by parents had a daytime sleep duration that was 1 h and 9.6 min less than that of toddlers bottle/breastfed fed to initiate sleep (*p* < 0.001). Daytime sleep duration was 1 h and 9.6 min less than in toddlers slept independently compared to toddlers breast/bottle fed to initiate sleep (*p* < 0.001) (Table 3).

**Table 3:** Multivariable linear regression models of number of nocturnal awakenings and daytime sleep duration

Sleep patterns	Number of nocturnal awakenings				Daytime sleep duration		
	β	95%CI	*p*		β	95%CI	*p*
In bed near parent	-1.496	-1.84∼-1.15	< 0.001		-1.16	-1.84∼-0.48	< 0.001
In bed alone	-1.691	-2.13∼-1.25	< 0.001		-1.394	-2.26∼-0.53	< 0.001
Being held	-0.803	-1.36∼-0.24	0.005		-0.363	-1.47∼0.75	0.521
Being rocked	-0.521	-1.39∼0.35	0.238		0.028	-1.69∼1.75	0.974

95% CI, 95% confidence interval; β, unstandardized regression coefficient

### Reliability analysis

A multivariable logistic regression model of nap consistency across the sleep pattern showed a significant effect of age category on nap consistency. The adjusted association between toddlers in the annual family income of 10,000 to 50,000 Renminbi (RMB) and the sleep pattern of being rocked consistency approached significance (adjusted odds ratio, 65.61; 95% CI, 1.12 ∼ 3833.12). The adjusted odds of having consistent sleep patterns in the annual family income lower than 10,000 RMB group compared to the annual family income higher than 200,000 RMB group was 4.52(95%CI,1.59∼12.852). The toddlers with a very serious sleep problem were more prone to be rocked to initiate sleep, and those with a small or little sleep problem were more probably to be in bed alone to initiate sleep(*p* < 0.05) (Table 4).

**Table 4:** Multivariable logistic regression model with sleep pattern as the dependent variable

**Independent variable**		**While feeding**				Being rocked				Being held				In bed alone	
	*Parameter Estimate (β)*	*OR(95%CI)*	*p*		*Parameter Estimate (β)*	*OR(95%CI)*	*p*		*Parameter Estimate (β)*	*OR(95%CI)*	*p*		*Parameter Estimate (β)*	*OR(95%CI)*	*p*
Annual family income	-1.42		0.41		-4.51	(∼)	0.23		-17.71	(∼)	1		0.69	(∼)	0.68
< 10,000 RMB	-0.53	0.592(0.141∼2.475)	0.47		3.16	23.459(0.19∼2893.736)	0.2		1.23	3.434(0.87∼13.553)	0.08		1.51	4.52(1.59∼12.852)	0.01
10,000 ~ 50,000 RMB	0.24	1.27(0.539∼2.994)	0.59		4.18	65.613(1.123∼3833.117)	0.04		0.36	1.431(0.433∼4.727)	0.56		0.4	1.489(0.613∼3.615)	0.38
50,000 ~ 100,000 RMB	0	0.998(0.436∼2.283)	1		-11.88	0.00(0∼.c)	0.99		0.15	1.162(0.386∼3.501)	0.79		0.45	1.567(0.723∼3.394)	0.26
> 100,000 RMB	Reference	1	.		Reference	1	.		Reference	1	.		Reference	1	.
Housing per capita area(m^2^)															
< 10	1.24	3.437(0.766∼15.415)	0.11		0.3	1.352(0.069∼26.54)	0.84		0.51	1.66(0.271∼10.176)	0.58		-1.2	0.302(0.042∼2.168)	0.23
10 ~ 30	0.71	2.025(0.989∼4.147)	0.05		-2.7	0.067(0.004∼1.121)	0.06		0.14	1.146(0.462∼2.843)	0.77		-0.37	0.69(0.362∼1.315)	0.26
> 30	Reference	1	.		Reference	1	.		Reference	1	.		Reference	1	.
Parenting experience (first-time caregiver)															
Stern	-1.82	0.162(0.017∼1.518)	0.11		1.27	3.575(0.003∼3926.427)	0.72		16.3	11940000(0∼.c)	1		-2.53	0.08(0.01∼0.761)	0.03
Natural	-1.21	0.297(0.028∼3.184)	0.32		3.64	38.25(0.044∼33280.164)	0.29		15.59	5923659.802(0∼.c)	1		-2.26	0.104(0.01∼1.098)	0.06
Multiple approaches	Reference	1	.		Reference	1	.		Reference	1	.		Reference	1	.
Screen time on different devices (min)															
< 30	0.92	2.518(0.622∼10.188)	0.2		-2.93	0.054(0.002∼1.853)	0.11		-0.84	0.43(0.077∼2.389)	0.34		1.16	3.193(0.365∼27.914)	0.29
30 ~ 60	-1.15	0.317(0.063∼1.588)	0.16		-20.15	0.000000001782(0∼.c)	0.98		-0.58	0.562(0.101∼3.14)	0.51		1.18	3.267(0.367∼29.042)	0.29
60 ~ 120	-0.35	0.706(0.13∼3.84)	0.69		-16.68	0.00000005714 (0∼.c)	0.99		-0.68	0.509(0.078∼3.337)	0.48		0.02	1.021(0.086∼12.192)	0.99
> 120	Reference	1	.		Reference	1	.		Reference	1	.		Reference	1	.
Sleeping position															
On his/her belly	-0.82	0.442(0.174∼1.12)	0.09		-2.5	0.082(0.001∼10.218)	0.31		-1.04	0.352(0.072∼1.712)	0.2		0.35	1.415(0.608∼3.291)	0.42
On his/her side	-0.95	0.385(0.198∼0.751)	0.01		0.47	1.593(0.225∼11.282)	0.64		0.02	1.018(0.429∼2.416)	0.97		-0.35	0.706(0.363∼1.372)	0.3
On his/her back	Reference	1	.		Reference	1	.		Reference	1	.		Reference	1	.
Sleeping arrangement															
Infant crib in a separate room	2.85	17.263(0.348∼855.306)	0.15		1.97	7.178(0.009∼5549.512)	0.56		-16.22	0.00000008991(0∼.c)	1		1.76	5.804(0.343∼98.16)	0.22
Infant crib in parents’ room	1.05	2.864(0.248∼33.088)	0.4		-19.1	0.000000005091(0∼.c)	0.99		-0.62	0.538(0.033∼8.808)	0.66		-0.11	0.894(0.174∼4.584)	0.89
In parents’bed	1.06	2.875(0.267∼30.904)	0.38		-0.44	0.647(0.01∼42.24)	0.84		-0.81	0.446(0.029∼6.91)	0.56		-1.06	0.348(0.071∼1.699)	0.19
Infant crib in room with sibling	2.41	11.177(0.359∼348.034)	0.17		-12.2	0.000005022 (0∼.c)	1		-15.82	0.0000001344(0∼.c)	1		2.13	8.44(0.848∼83.987)	0.07
Other	Reference	1	.		Reference	1	.		Reference	1	.		Reference	1	.
Sleep problem															
A very serious problem	1.86	6.414(0.967∼42.526)	0.05		6.85	947.715(4.234∼212145.014)	0.01		-17.47	0.00000002586(0.00000002586∼0.00000002586)	.		-17.78	0.00000001898(0∼.c)	1
A small problem	0.3	1.344(0.572∼3.157)	0.5		0.8	2.23(0.116∼42.883)	0.6		-0.11	0.897(0.23∼3.501)	0.88		-1.86	0.156(0.033∼0.744)	0.02
A little problem	-0.31	0.731(0.334∼1.597)	0.43		1.95	7.055(0.475∼104.783)	0.16		0.52	1.676(0.698∼4.027)	0.25		-1.24	0.291(0.126∼0.668)	0
Not a problem at all	Reference	1	.		Reference	1	.		Reference	1	.		Reference	1	.

Note: Taking parental accompaniment to sleep as a reference category; residence, parenting style, mother’s education level, feeding method, frequency of baby night awakenings, and sleeping bed arrangements are considered as control variables

## Discussion

Infancy is the fastest growing period of physical and psychological development in a person’s life, good sleep may have an important impact on the physical growth, psychological and behavioral development of infants. The growth in length of toddlers is associated with extended sleep and increased naps [6]. The sleep routines of toddlers and their parents were disturbed during the COVID-19 pandemic, especially COVID-19-related lockdowns. Interestingly, diverse alterations in sleep patterns among pediatric populations in different countries have been observed during the COVID-19 pandemic, as highlighted in pertinent literature, and there is no consistent expectation of worsening sleep patterns. In comparison to the 2018 sample, preschoolers (aged 4–6 years) in Southwest China exhibited altered sleep patterns before the COVID-19 outbreak, including later wake times and bedtimes, shorter nap durations, extended nocturnal sleep, and reduced sleep disturbances [7]. Research carried out during the complete lockdown in Italy, focusing on children aged 2–5 years, indicated noticeable delays in both wake-up and bedtime routines. Nevertheless, the proportion of children with some sleep difficulties (i.e., SDSC > 39) remained constant, increasing from 41.46% before the lockdown to 44.72% during the lockdown [14]. An Israel research explored the possible negative implications of the COVID-19 pandemic on sleep for both children aged 6 to 72 months and their mothers. However, data showed that approximately 30% of mothers reported a decrease in the children’s sleep duration and quality, and also mothers reported positive changes [15]. A meta-analysis encompassing children and adolescents from diverse Asian countries, South America, and European countries demonstrated that at least 21.3% of children experienced sleep disturbances during COVID-19 pandemic [16]. As reported in the research by Cassanello et al. [8], longer sleep latencies (> 30 min) ware observed in more infants and toddlers (with a median of 33.9% versus 12.3%). Before and under the COVID-19 outbreak lockdown, the proportions of children whose sleep perceived as problematics were 39.4% and 44.1%. This study identified that feeding to sleep and cuddling to sleep significantly reduced the length of nighttime sleep among the toddlers during the pandemic of COVID-19. In our study, 331(67.1%) initiated sleep accompanied by parents, 67(13.6%) slept independently, 59(12.0%) were breast fed/bottle fed to initiate sleep, 27(5.5%) were held and 9 (1.8%) rocked during COVID-19 pandemic. Thus, although the proportion of infants and children of different ages who fall asleep poorly varies in different places, poor sleep patterns are still widespread.

Poor sleep pattern is defined as the inability to fall asleep alone and relying on behaviors such as nursing, patting and rocking that are not for feeding purposes. The present study showed that different sleep-initiated patterns influenced the length of night sleep and the number of night awakenings of toddlers, among who were fed, rocked, and held to sleep were significantly more frequent than those who fell asleep on their own. Regular nighttime breast feeding, excessive viewing, or lack of general knowledge about child sleep hygiene (e.g., mistaking vocalizations, sport activities, and facial expressions that occur in the baby’s light sleep state as signs that the baby is awake and picking the baby up) may artificially increase the number of infants and toddlers waking up at night [17]. Additionally, sleep initiation with bottle feeding/breast feeding, rocked and cuddled may all exhibit certain poor bedtime soothing behaviors, which interfere with the normal development of young children’ sleep rhythms, resulting in longer sleep latency, more nighttime awakenings, and poorer sleep [8, 18]. A multi-center cross-sectional survey on the dose-dependent relationship between sleep outcomes and bedtime routines in infants and young children from mainland China indicated that 48.5% (632/1304) children had not established regular sleep routines reported by the parents. The more regular sleep routines were associated with earlier bedtime, fewer night awakenings, shorter sleep onset latency and duration of nighttime waking, and longer the sleep duration. For example, people who had a bedtime routine “every night” had significantly longer nighttime sleep than those who “never” had a bedtime routine[9.5(95%CI: 9.4–9.6) vs 0.8.9(95%CI: 8.6–9.3), t = 3.345, *p* = 0.001] [19]. Sleep initiation patterns in young children were associated with extended use of electronic devices, and young children who are exposed to electronic screens every day have a higher probability of being breast fed/bottle fed to sleep [20]. The fact that screen time viewing replaces other sleep-friendly activities [21], such as parent-child interactions or exercise duration, and excessive screen time can trigger sleep problems [22]. It is estimated that for every hour of screen use, sleep is delayed by 5–10 min [21]. For this of reason, the American Academy of Pediatrics recommends not using them at least 1 h before bedtime [23]. Our findings show that a higher probability of needing to be rocked to sleep at bedtime in toddlers exposed to electronic screens on a daily basis, which is hypothesized to be related to an increase in sleep latency, where bright light may lead to cognitive and emotional activation [24], producing a state of heightened arousal [25], reducing melatonin production [26], and a delay in sleep latency and the need for alternative ways to help falling asleep. During the COVID-19 pandemic, limited outdoor activities, lack of interaction with children of the same age, and excessive electronic screens may result in delayed sleep, more sleep problems, and decreased sleep quality compared to weekends and holidays. These may further have a negative impact on physical and mental health, and therefore it requires attention and intervention.

Frequent nocturnal awakening is as one of the manifestations of circadian rhythm disturbance. The incidence of problematic nocturnal awakenings is higher in our infants and young children compared to overseas children of the same age [17]. In the current study, the 1–2, and 2–3 rates of toddlers waking up more than once a night was 64.6% and 67.2%, respectively. In addition, parental bedtime habits and toddlers’ sleep patterns are intricately linked, displaying a high degree of chronicity in sleep problems that are influenced by previous sleep behaviors and sleep arrangements. Clinically, when parents are counseled about co-sleeping their infants, the quality of the mother’s sleep should be taken into account. A cross-sectional survey conducted in 2019 (before the outbreak of COVID-19) from six provinces in China indicated that the bed-sharing practice ranged from 69.9 to 78.3%, which was very prevalent at any age. Most young children before age 12 months fell asleep while being rocked/held or feeding. By 35 months of age, 62.4% of children slept in the bed alongside their parents. The most popular explanation for bed-sharing were breast-feeding/bottle-feeding, and difficulty falling asleep and frequent night awakenings among the children were significantly impacted by parental involvement [27]. Good sleep quality is associated with more regular sleep routines [19].

Despite the fact that bed-sharing and parental involvement are still widely practiced among young children in China, on the contrary, our study found young children falling asleep along with parents have less nighttime awakenings during the COVID-19 pandemic. Nor was there an association between sleep and bed-sharing. Bed-sharing remained an independent and graded predictor of nocturnal awakenings and shortened sleep duration, even after controlling for preceding sleep conditions [28]. Irregular and later parental bedtime routines worsted infants and toddlers sleep [29]. Nonetheless, an important finding revealed that children under 36 months of age in Hong Kong who were able to fall asleep independently exhibited fewer instances of sleep awakenings and enjoyed longer nocturnal sleep duration, whereas sleep location did not show the same association. This is especially important for families with restricted living space who cannot avoid parents/children sharing beds or rooms [29]. At present, the proportion of regular sleep patterns among young children in mainland China is still low, and breastfed children mostly rely on breast fed/bottle fed to initiate sleep, while most older toddlers fall asleep mainly in the company of their parents. Further research is required to discern the potential advantages of how bed-sharing interacts with infant care practices beyond sleep [20]. Furthermore, the negative impact of insufficient and inadequate sleep on children’s physical and mental health are indisputable, as are the effects on cognitive functions, academic achievement and performance, all of which are higher risk factors for children in low- and middle-income countries. Behavioral interventions for mothers and young children that are inexpensive and do not require specialized training can reduce the risks faced by these children and thus help prevent future problems [30].

A limitation that is the use of single assessments of sleep in young children, including parent-reported sleep questionnaires. Indeed, multiple assessments of sleep in young children, including parent-reported sleep diaries and actigraphy-based assessments should be considered. Given the sample size, this is a cross-sectional observation of 12- to 35-month-old children in Fujian Province only. And a larger sample size including more ethnic/racial groups are needed in more studies, especially longitudinal studies. There are, however, the causal relationship between sleep quality and sleeping patterns (bedtime, sleep duration), sleep behaviors still need to be assessed by further research. However, it is important to note that there may be some differences in sleep patterns between the during and after COVID-19 pandemic. While our study provides valuable insights into the potential impact of the COVID-19 pandemic on children’s sleep, further research is needed to follow up the sleep of children during and after the COVID-19 pandemic in the following study for comparison.

## Conclusions

In summary, most infants and toddlers in the Southeast of Mainland China initiated sleep accompanied by parents and tend to have electronic media exposure before bedtime during the COVID-19 pandemic. Increased waking at night may be associated with sleep initiation with breast-feeding/bottle-feeding. Therefore, pediatric practitioners in primary community hospitals should pay attention to the education and promotion of sleep hygiene and parenting knowledge of young children to avoid the formation of poor sleep hygiene habits.

## Data Availability

The datasets utilized and analyzed in this study can be obtained upon request from the corresponding author.
